# Diagnostic and therapeutic challenges in colonic eosinophilic abscess complicated with eosinophilic vasculitis: a case report and literature review

**DOI:** 10.3389/fmed.2025.1587226

**Published:** 2025-07-08

**Authors:** Na Ding, Jianru Zhao, Feng Tian, Hui Li

**Affiliations:** Department of Gastroenterology, Shengjing Hospital of China Medical University, Shenyang, Liaoning, China

**Keywords:** colonic eosinophilic abscess, eosinophilic vasculitis, eosinophilia, histopathological analysis, hypereosinophilic syndrome

## Abstract

This case report and literature review present a 52-year-old male with a sigmoid eosinophilic abscess (EA) complicated by eosinophilic vasculitis (EoV), resulting in thrombosis and intestinal perforation. The patient presented with acute abdominal pain and melena. Laboratory tests revealed marked eosinophilia, elevated D-dimer levels, and a white blood cell count consistent with systemic inflammation. Contrast-enhanced computed tomography (CECT) identified extensive vascular thrombosis and bowel wall compromise, necessitating surgical intervention. Histopathological examination confirmed the diagnosis of EA with associated eosinophilic infiltration in the intestinal wall. This case highlights the diagnostic and therapeutic challenges of eosinophilic disorders, particularly the need for awareness of their potential complications. It also emphasizes the importance of timely intervention in managing such rare conditions. Through a review of the relevant literature, we further discuss the characteristics, differential diagnosis, and management strategies for EA and EoV.

## Introduction

Eosinophilic disorders are a diverse group of pathological conditions characterized by an abnormal increase in eosinophils in the blood or tissues. These disorders can be classified based on the peripheral absolute eosinophil count (AEC) into mild eosinophilia (0.5–1.49 × 10^9^/L), moderate hypereosinophilia (1.5–5.0 × 10^9^/L), and severe hypereosinophilia (greater than 5.0 × 10^9^/L) (1). Hypereosinophilic syndrome (HES) is diagnosed when hypereosinophilia (HE) is associated with tissue or organ damage (2, 3). HES can be further classified into familial (hereditary), idiopathic, secondary (reactive), and primary (clonal, neoplastic) subtypes (3). Secondary HES, the most common variant and can result from infections, allergies, drug reactions, autoimmune disorders, and neoplastic conditions (4).

HES can affect various organ systems, including the skin, gastrointestinal tract, pulmonary and upper respiratory tracts, as well as the cardiovascular and central nervous systems (5). Gastrointestinal involvement in HES often presents as eosinophilic esophagitis, gastroenteritis, or colitis, characterized by symptoms such as abdominal pain, vomiting, diarrhea, and difficulty swallowing (6). Based on the depth of eosinophilic infiltration, eosinophilic gastroenteritis is classified into three types: mucosal, muscular, and serosal (7). EA, a rare condition associated with HES, is characterized by eosinophilic infiltration forming tumor-like lesions in affected tissues. It was first described by Albot et al. (8) in the context of eosinophilic cholecystitis, characterized by dense eosinophilic infiltration in the gallbladder wall. EA can arise from diverse underlying causes, such as parasitic infections, allergic diseases, tumors, and other eosinophilia syndromes (9). While EA most commonly affects the liver due to its strategic anatomical location and rich blood supply, other sites such as the lungs, esophagus, bile ducts, ribs, mediastinum, and lymph nodes can also be affected (10). However, gastrointestinal involvement in EA has not been previously reported. Here, we present a case of a Chinese farmer with a sigmoid mass complicated by widespread thrombosis and sigmoid perforation. Postoperative histopathology revealed the diagnoses of EA and EoV. This case highlights the diagnostic and therapeutic challenges of eosinophilic disorders, underscoring the importance of timely intervention.

## Case description

**Figure 1:**
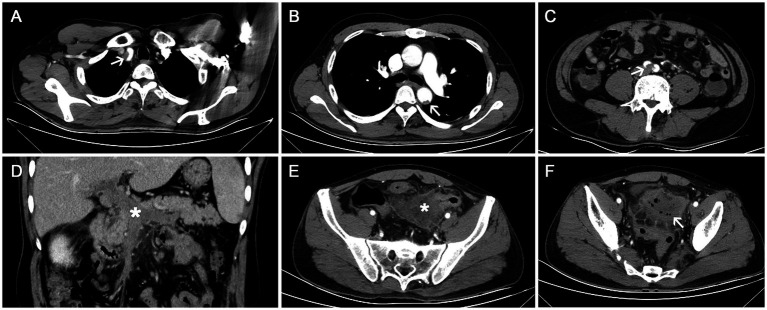
Contrast-enhanced computed tomography (CECT) scan. **(A–C)** Thrombosis in the brachiocephalic trunk, thoracic aorta, and bilateral iliac arteries (white arrows); **(D)** Thrombosis in the portal vein system (white star); **(E,F)** Wall thickening of the sigmoid colon with an abscess (white star) and extraluminal gas (white arrow), indicating perforation.

**Figure 2:**
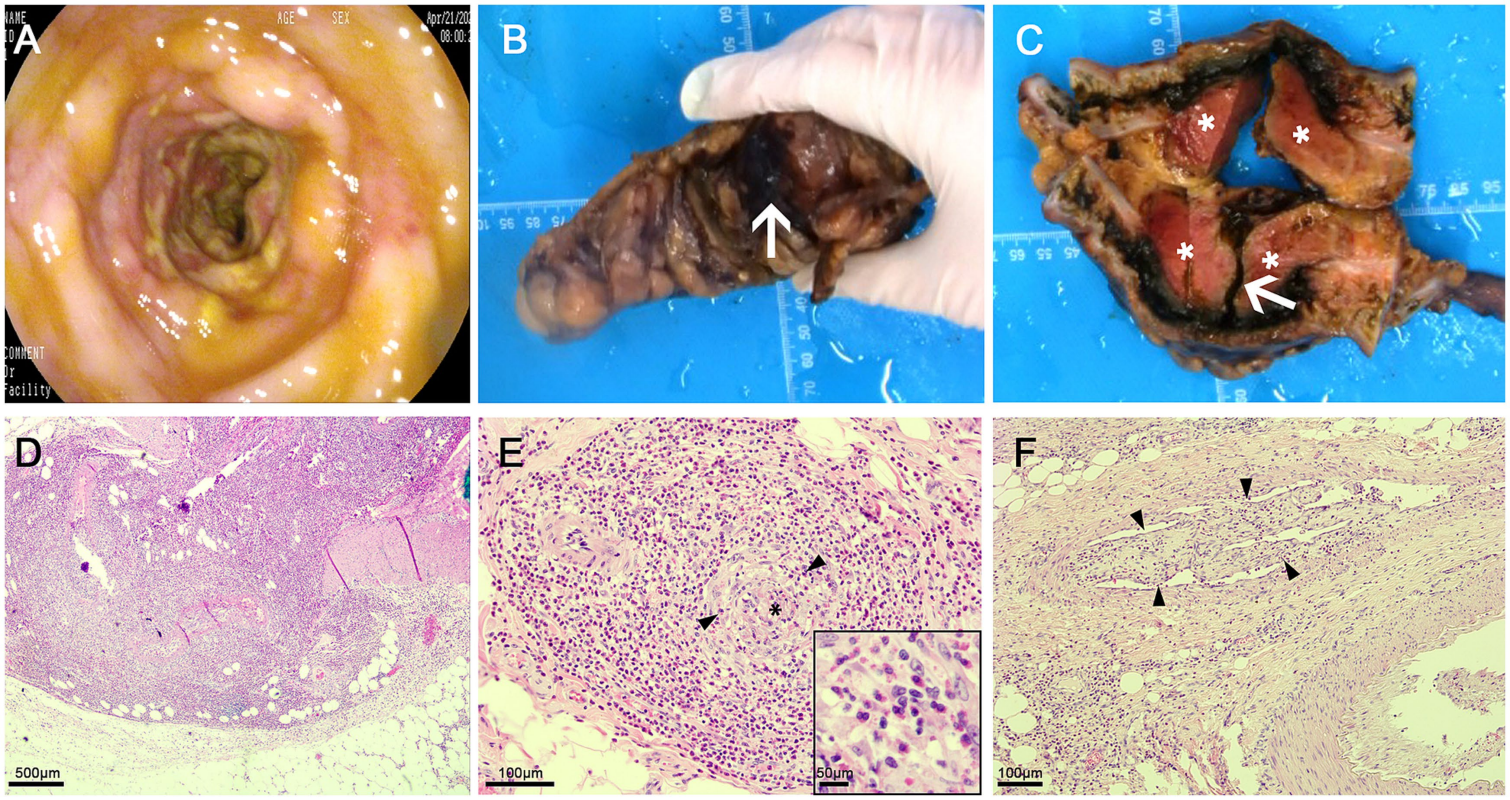
**(A)** Sigmoid colonoscopy revealing edematous mucosa and patchy ulcers; **(B)** Perforation of the sigmoid colon with purulent exudate on the surface (arrow); **(C)** A 6 × 3 cm mass observed outside the intestinal wall adjacent to the perforation following incision, appearing as dark red necrosis (asterisk), along with a deep ulcer in the sigmoid colon (arrow). **(D)** Eosinophilic abscess: A dense infiltrate of mature eosinophils is present within the mass outside the intestinal wall, associated with coagulative necrosis of the corresponding intestinal wall; **(E)** Eosinophilic vasculitis: Numerous eosinophils are seen infiltrating around and within the walls of submucosal small veins (black arrow heads), leading to vascular occlusion (black asterisk); **(F)** Thrombus formation in the serosal layer veins, with organization and recanalization (black arrow heads), and eosinophil infiltration surrounding the vessels, within the walls, and in the vascular lumen.

A 52-year-old male patient presented with the sudden onset of lower abdominal pain lasting 8 days, accompanied by melena. He had no significant medical history, including no allergies or skin diseases. Physical examination revealed localized tenderness in the left lower quadrant without muscle rigidity. Laboratory tests showed a white blood cell count of 16.4 × 10^9^/L (reference range: 4.0–9.0), eosinophil count of 3.8 × 10^9^/L (reference range: 0.04–0.49), platelet count of 41 × 10^9^/L (reference range: 120–300), elevated D-dimer level of 10,781 μg/L, and a C-reactive protein level of 128.5 mg/L (reference range: <5). Urinalysis was unremarkable. Tumor markers and IgE were within normal limits. Antineutrophil cytoplasmic antibodies (ANCA) testing was negative. Given the elevated peripheral eosinophil count, repeated fecal parasite examinations were conducted to exclude parasitic infection. Pulmonary function tests were normal, making asthma an unlikely diagnosis. Bone marrow examination and flow cytometry were performed. The bone marrow analysis revealed an AEC of 2.04 × 10^9^/L. Flow cytometry showed that eosinophils constituted 17.6% of the cellular composition, with no evidence of hematologic malignancy. CECT scans disclosed thrombosis in the brachiocephalic trunk, thoracic aorta, abdominal aorta, bilateral iliac arteries, and the portal vein system (Figures 1A–D). There was also bowel wall thickening, an abscess, and extraluminal gas in the sigmoid colon, indicating perforation (Figures 1E,F). Sigmoid colonoscopy revealed edematous mucosa and patchy ulcers (Figure 2A). The patient underwent partial resection of the sigmoid colon with a protective proximal colostomy. Intraoperatively, a 6 × 3 cm mass adherent to the serosal surface of the resected colon was identified (Figures 2B,C). Hemopurulent ascites were present in the abdominal cavity, with a perforation in the middle segment of the sigmoid colon and localized encapsulation. The jejunum, located 20 cm from the ligament of Treitz, exhibited dark red discoloration and edematous thickening of the intestinal wall, suggesting venous return obstruction. Histopathological examination of the mass revealed a dense infiltration of mature eosinophils with coagulative necrosis in the adjacent intestinal wall, confirming EA (Figures 2D,E). Furthermore, eosinophilic infiltration was observed within small vessels of the mesangial area, serosa, muscular layer, and submucosal layer of the intestinal wall, accompanied by thrombosis, organization, and recanalization within the intravascular lumen, consistent with EoV (Figure 2F). Few eosinophilic infiltration was observed at the resected intestinal margins. Based on these pathological findings, the diagnosis of colonic serosal eosinophilic abscess complicated by vasculitis and thrombus formation, leading to intestinal wall necrosis and perforation, was established.

**Figure 3:**
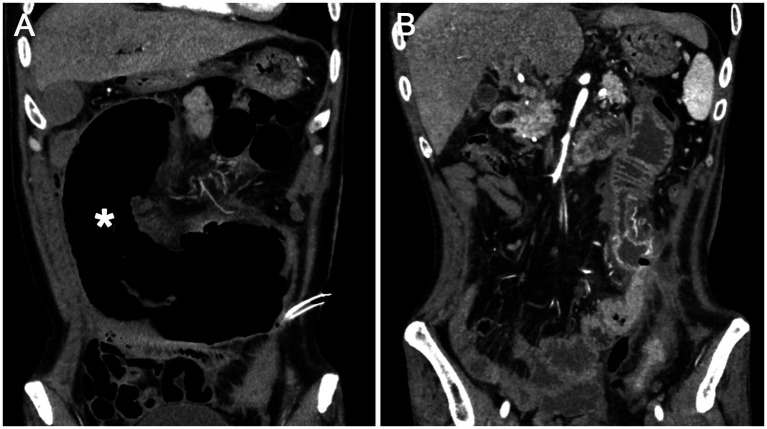
**(A)** Dilation of the jejunum (asterisk) indicating mechanical intestinal obstruction and ischemia; **(B)** Six months post-surgery, improvement in jejunal obstruction is evident.

**Figure 4:**
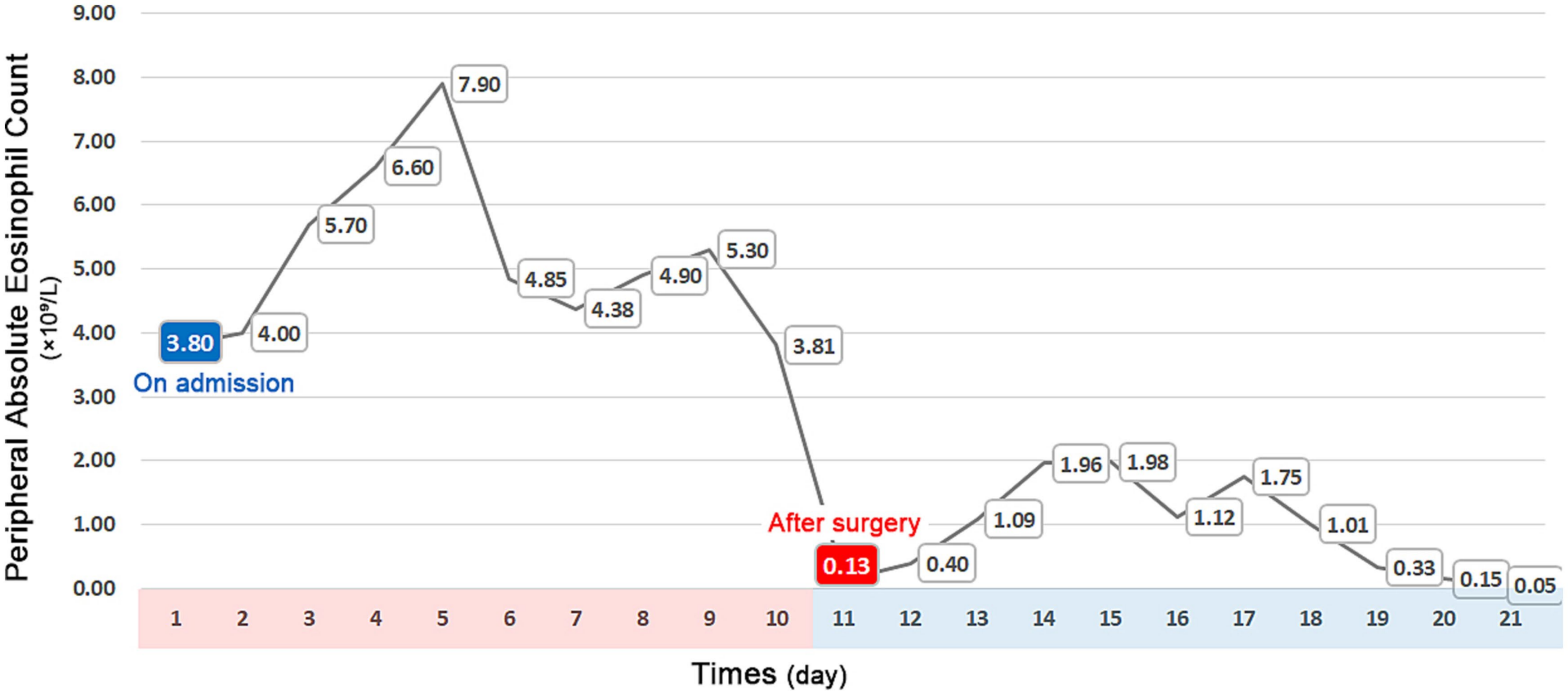
Timeline of peripheral absolute eosinophil counts, showing a significant increase upon admission and normalization following surgery.

The patient experienced significant clinical deterioration, complicated by disseminated intravascular coagulation (DIC) and persistent abdominal drain bleeding, which contraindicated the initiation of steroid therapy. He required transfer to the intensive care unit for multiorgan support. Repeat abdominal CT demonstrated severe small bowel obstruction (Figure 3A). Despite these complications, postoperative eosinophil levels normalized, and the patient showed clinical improvement with anticoagulant therapy, anti-infective agents, and supportive care. Since there was no evidence of active inflammation, steroid therapy was not administered. A 6-month follow-up CT scan showed resolution of thrombi in the brachiocephalic artery and pulmonary artery, and a reduction in multiple mural thrombi in the aorta and its branches (Figure 3B). Although extensive thrombi remained in the portal vein, superior mesenteric vein, and splenic vein, with cavernous transformation, significant improvement in jejunal ischemia and obstruction was noted. At the 2-year follow-up, the patient experienced complete resolution of symptoms and maintained normalized eosinophil and platelet counts. Figure 4 illustrates the timeline of the AEC before and after surgery.

## Discussion

This case report highlights a rare and complex presentation of sigmoid colon EA complicated by EoV and subsequent thrombosis, which led to intestinal perforation. This is the first reported case of colonic EA occurring without concurrent eosinophilic gastroenteritis. We also review the literature on the characteristics of EA.

Eosinophils originate in the bone marrow from GATA-1-binding factor 1 (GATA-1)–positive granulocyte-monocyte progenitors and can be stimulated by interleukins (IL-3, IL-5) and granulocyte/macrophage colony-stimulating factors. They are released into the peripheral blood and rapidly migrate to specific target organs (11, 12). A variety of chemokines, including stromal cell–derived factor-1 (SDF-1), CCL5 (RANTES), CCL11 (eotaxin-1), CCL24 (eotaxin-2), CCL26 (eotaxin-3), and platelet-activating factor (PAF), can induce eosinophil migration, activation, and chemotaxis (13–15). EA formation occurs primarily through two mechanisms: (1) the release of cytotoxic granule proteins and inflammatory lipid mediators during eosinophilia, leading to organ damage; and (2) direct parasitic invasion resulting in focal hepatic lesions (16–18). Eosinophil granules contain cationic cytotoxic proteins, such as eosinophil cationic protein (ECP), major basic protein (MBP), eosinophil peroxidase (EPO), and eosinophil-derived neurotoxin (EDN). These granule proteins can directly damage host tissues by disrupting cell membranes and inducing cell death. Moreover, these proteins can predispose to thrombosis by acting as platelet agonists, increasing vascular permeability, stimulating activation of factor XII, and reducing the anticoagulant actions of heparin (3, 19). Extensive eosinophilic tissue infiltration and degranulation can cause massive venous and arterial thrombosis, potentially leading to consumptive coagulopathy and subsequent DIC, which may result in fatal bleeding complications (20).

The clinical manifestations of EAs depend on the affected organ and etiology, varying in severity. EAs often present asymptomatically and may be incidentally detected on routine abdominal CT, leading to frequent misdiagnoses as primary or metastatic malignancies. Symptoms such as fever, abdominal pain, or jaundice may occur due to parasitic infections or biliary duct involvement (21–23). EAs secondary to eosinophilic gastrointestinal tract infiltration have been documented, particularly in eosinophilic esophagitis, presenting as paraesophageal, mediastinal, or esophageal wall abscesses, with or without pre-existing trauma (24–26). Unlike our case, few eosinophilic infiltrations were observed at the resected intestinal margins, and postoperative serum eosinophil levels returned to normal. These findings indicated that the EA was not secondary to eosinophilic enteritis.

Most EA cases reported in the literature involve the liver, presenting with non-specific imaging findings. CT scans typically show small, round, or oval lesions with ill-defined margins, isoattenuation on unenhanced CT scans, hyperattenuation during the hepatic arterial phase, and hypoattenuation in the portal venous phase (27). MRI findings include low signal intensity on T1-weighted images and high signal intensity on T2-weighted images, with heterogeneous enhancement during the hepatic arterial phase and low signal intensity in the portal venous phase (28, 29). Ultrasound examinations usually reveal small, poorly defined, round or oval hypoechoic nodules (30, 31). Pathologically, EAs exhibit dense eosinophilic infiltration, often accompanied by other inflammatory cells, such as lymphocytes and histiocytes, which cause focal necrosis and hepatocellular damage in the liver, particularly in the periportal areas (26). In eosinophilic gastrointestinal diseases, microabscesses, dense eosinophilia, eosinophil degranulation, and epithelial inflammation (e.g., basal zone hyperplasia, rete peg elongation) are described (32, 33). Visceral larva migrans, caused by second-stage larvae of nematodes like Toxocara canis migrating through human viscera, can lead to inflammatory responses with eosinophilic infiltration, resulting in eosinophilic granulomas or, in some cases, EAs formation (9). Focal eosinophilic infiltration in the liver is characterized by periportal and lobular infiltration, with preservation of normal histologic architecture (30, 34, 35). Eosinophilic granuloma typically features central necrosis and a mixed inflammatory cell infiltration, primarily comprising eosinophils and epithelioid histiocytes. The term EA is used when there is massive eosinophilic infiltration and destruction of liver parenchyma with inflammation (10, 23).

The differential diagnosis of EA includes parasitic infection, malignancy, eosinophilic granulomatosis with polyangiitis (EGPA), and Kimura’s disease (KD). EAs are frequently observed in parasitic infections, such as visceral larva migrans, which can lead to local eosinophilic infiltration, thrombosis, and necrosis. However, in this case, the negative fecal exam, histopathology, and postoperative eosinophil normalization in this case argue against this etiology. Differentiating EAs from malignancies is challenging. On MRI, liver metastases often show sharply defined margins, irregular shapes, and arterial nodular enhancement with washout in the portal venous phase and delayed capsular enhancement. In contrast, most EAs are isointense on both the portal and equilibrium phases (36). However, differentiating arterial-enhancing eosinophilic abscesses or focal eosinophilic infiltration from hepatocellular carcinoma or hypervascular hepatic metastases remains difficult (37). Primary tumors may also induce eosinophilic abscesses, mimicking hepatic metastases (38). Several reports highlight the relatively high incidence of co-occurrence between focal eosinophilic liver disease and malignant conditions (29, 36, 39, 40), underscoring the importance of histopathological examination for differentiation. KD is a rare, benign, chronic inflammatory disorder presenting with slowly enlarging, non-tender subcutaneous masses, often associated with regional lymphadenopathy or salivary gland involvement (41). Histologically, KD is characterized by lymphoid follicular hyperplasia with germinal center enlargement, eosinophilic infiltration, accumulation of eosinophilic microabscesses, and postcapillary and venular hyperplasia, surrounded by circular collagenous fibrous deposition and varying degrees of fibrosis (42). EGPA should be considered in the differential diagnosis of EA and vasculitis. The disease is characterized by asthma, eosinophilia, and small vessel vasculitis. According to the 2022 ACR-EULAR classification criteria, a diagnosis requires a score of ≥6 based on features including maximum eosinophil count, obstructive airway disease, nasal polyps, and ANCA positivity (43). Histopathologically, EGPA features eosinophilic infiltrates, necrotizing vasculitis, and extravascular granulomas (44–46). However, this patient lacked typical clinical features (asthma, nasal polyps) and key pathological findings (eosinophilic granulomas). Serological ANCA testing was also negative. Collectively, these findings make EGPA an unlikely diagnosis. Other rare conditions to consider in the differential diagnosis of eosinophilia include drug reaction (e.g., DRESS syndrome), hematologic malignancies (e.g., systemic mastocytosis), autoimmune diseases (e.g., IgG4-related disease), and immunodeficiency disorders (e.g., hyper-IgE syndrome and IgA deficiency). DRESS can present with eosinophilia, thrombosis, and organ damage. However, the lack of drug exposure history and postoperative improvement without steroids made this diagnosis improbable. Primary immunodeficiency was considered given the thrombosis and infection tendency, but the patient’s normal IgE levels and lack of recurrent infections were inconsistent. In this case, the EA was exclusively located in the sigmoid colon with no evidence of systemic involvement. The rapid normalization of eosinophilia post-resection without immunosuppression distinguishes this case from autoimmune-associated eosinophilic disorders or hematologic malignancies, which typically require ongoing therapy.

EoV and thromboembolic complications are common and severe in HES, potentially leading to stroke, intracavitary cardiac thrombi, and vascular thrombosis (14). EoV is characterized by eosinophilic infiltration into blood vessel walls, primarily affecting small vessels. These eosinophils release cytotoxic proteins that promote inflammation, fibrosis, and thrombosis, thereby damaging the endothelial cells that line the blood vessels. This can impair blood flow and lead to tissue ischemia. According to Lefèvre et al. (47), EoV was identified in 12% of HES patients. Thrombosis is a significant complication associated with EoV, affecting various organs and systems, including the cardiac, intra-abdominal, cerebral, cutaneous, and deep venous thromboses (48–53). Studies suggest approximately 25% of EoV patients may experience thromboembolic events, with mortality rates ranging from 5 to 10%, emphasizing the seriousness of EoV and the importance of timely and effective treatment (51).

Anti-parasitic therapy is essential for EAs caused by parasitic infections. Studies indicate that empirical anti-parasitic medications may lead to faster improvement in imaging for patients with unknown causes, suggesting that many such cases may be related to parasitic infections (54). Surgical resection is recommended when malignancy is suspected, as it can provide a definitive diagnosis and alleviate symptoms. Tailored subsequent therapy may be required, potentially involving antibiotics or antiparasitic treatment for positive cultures or steroids for refractory conditions, but lifelong treatment is generally unnecessary. Shanti et al. (10) documented two cases of EA presenting as bile duct masses, where patients recovered uneventfully following surgery without additional medications. Shigematsu et al. (55) described a case of multiple hepatic EAs associated with colon cancer. After resection of the sigmoid colon cancer, there was rapid normalization of peripheral eosinophilia and complete radiological resolution of hepatic lesions within 6 months. EoV is responsive to corticosteroid therapy, with approximately 40% of patients experiencing no recurrence following first-line therapy. However, some patients may require low-dose corticosteroid maintenance or combined immunosuppressive treatment (47). Huang et al. reported a case of a patient presenting with extensive thrombosis in the liver and spleen. Pathology from a colostomy revealed “ischemic colitis with EoV, “affecting medium-sized blood vessels and the surgically resected margin of the intestine. However, on the ninth day post-surgery, the patient exhibited peritonitis symptoms coinciding with an elevated blood eosinophil count, necessitating long-term remission maintenance with hormonal and immunosuppressive therapy (56). In our case, the patient’s serum eosinophil level was persistently elevated preoperatively but rapidly normalized following surgical resection of the EA. This clinical course suggests that the EA in the sigmoid colon served as the “initiator” of the eosinophilia. The EA likely infiltrated the intestinal wall and mesenteric small vessels, inducing vasculitis and intravascular thrombosis. These pathological changes led to localized ischemia and necrosis of the sigmoid colon, ultimately resulting in perforation. The EA possibly released many eosinophils into the peripheral circulation, causing widespread vascular damage, secondary thrombosis and embolism, and subsequent multi-organ ischemia. However, the etiology of EA formation in this patient remains unclear. Although literature reports suggest that EA is often associated with parasitic infections, no evidence of parasitic infection was identified in this case.

## Conclusion

This case report highlights the successful management of a rare and complex presentation of HES with sigmoid EA and EoV, complicated by widespread thrombosis and DIC. It emphasizes the critical role of timely treatment, including surgical intervention for obtaining pathological confirmation, in achieving a definitive diagnosis and guiding effective therapy for eosinophilia cases with obscure etiologies and localized lesions. It underscores the importance of considering rare conditions such as EA and EoV in patients presenting with unexplained eosinophilia and gastrointestinal symptoms. Clinicians should recognize the potential for severe complications in patients with HES and EA, and prompt detection and treatment are essential for improving outcomes.

## Data Availability

The original contributions presented in the study are included in the article/supplementary material, further inquiries can be directed to the corresponding author.
